# Self-evaluative reliability: a missing safety property in adaptive clinical AI governance

**DOI:** 10.3389/frai.2026.1973339

**Published:** 2026-10-01

**Authors:** Rasit Dinc, Nurittin Ardic

**Affiliations:** 1 INVAMED Medical Innovation Institute, New York, NY, United States; 2 Med-International UK Health Agency Ltd., Warwickshire, United Kingdom

**Keywords:** adaptive clinical AI, AI governance, good machine learning practice (GMLP), medical device regulation, post-deployment monitoring, predetermined change control plan (PCCP), self-evaluative reliability

## Abstract

Adaptive clinical AI governance is shifting from static pre-market approval processes toward continuous post-market oversight, creating circumstances in which AI-generated self-evaluation may contribute to post-deployment safety assurance. Existing regulatory approaches, such as Predetermined Change Control Plan and Good Machine Learning Practice principles, address monitoring, modification, and performance drift but do not explicitly define the reliability of AI-generated self-evaluation as a distinct validation objective. Emerging studies of AI-based clinical evaluation show substantial variability in agreement across models, evaluators, tasks, and evaluation criteria. Although this evidence stems largely from AI-as-evaluator rather than from direct self-evaluation, it demonstrates why evaluative reliability should not be inferred from general model performance. We define self-evaluative reliability (SER) as the consistency and dependability with which an AI system assesses the quality, uncertainty, or limitations of its own outputs under predefined evaluation conditions. We propose SER as a distinct safety property that should be independently validated when self-evaluative judgments contribute to monitoring, escalation, adaptation, or continued deployment. Rather than introducing a novel regulatory framework, SER extends established principles of reproducibility and reliability to AI-generated evaluations. Establishing this validation before self-evaluation becomes load-bearing could strengthen the safety and transparency of adaptive clinical AI governance.

## 1 Introduction

### 1.1 The hidden assumption

Clinical artificial intelligence (AI) regulation is entering a new phase in which approved systems may evolve after deployment, rather than remain functionally static. Recent regulatory and international guidance illustrate this shift. The United States Food and Drug Administration (FDA) has issued final guidance on Predetermined Change Control Plans (PCCP) for AI-enabled device software functions, while the International Medical Device Regulators Forum (IMDRF) has published Good Machine Learning Practice (GMLP) guiding principles for medical device development (International Medical Device Regulators Forum (IMDRF), 2025; U.S. Food and Drug Administration (FDA), 2025). These documents provide recommendations and principles for managing AI-enabled medical devices across modification, validation, monitoring, and lifecycle processes. Post-deployment monitoring is therefore becoming increasingly important for detecting changes in system performance and informing decisions about modification, retraining, or continued use (Keyes et al., 2026).

As clinical AI systems increasingly acquire agentic capabilities, AI-generated self-evaluation may become a useful input for post-deployment safety assurance. For instance, a system might identify uncertainty, detect inconsistencies, or signal that its outputs require additional review. Such capabilities could support monitoring, escalation, or adaptation processes without replacing independent human oversight. However, before self-evaluative signals are incorporated into safety-critical processes, a more fundamental question arises: How reliably can an AI system evaluate the quality and limitations of its own outputs? Current adaptive governance frameworks do not explicitly designate self-evaluative reliability (SER) as a distinct validation objective.

Emerging evidence from AI-based clinical evaluation demonstrates why this distinction matters. Studies of large language model (LLM)-based clinical evaluation have reported substantial disagreement between independent AI evaluators and model-dependent differences when LLM judges are compared with human evaluators (Byun et al., 2026; Williams et al., 2026). Although these studies examine AI-as-evaluator rather than self-evaluation directly, they challenge the broader assumption that evaluative competence can be inferred from general model capability.

In this Perspective, we define self-evaluative reliability (SER) as the consistency and dependability with which an AI system can assess the quality, uncertainty, or limitations of its own outputs under predefined evaluation conditions. We propose that SER should be treated as a measurable safety property that requires explicit validation when self-evaluation contributes to post-deployment monitoring, escalation, or adaptation. It should not be assumed to arise as a by-product of overall model competence. Figure 1 illustrates this conceptual shift and positions SER as a validation objective within the post-deployment assurance pathway (see Box 1).

**Figure 1:**
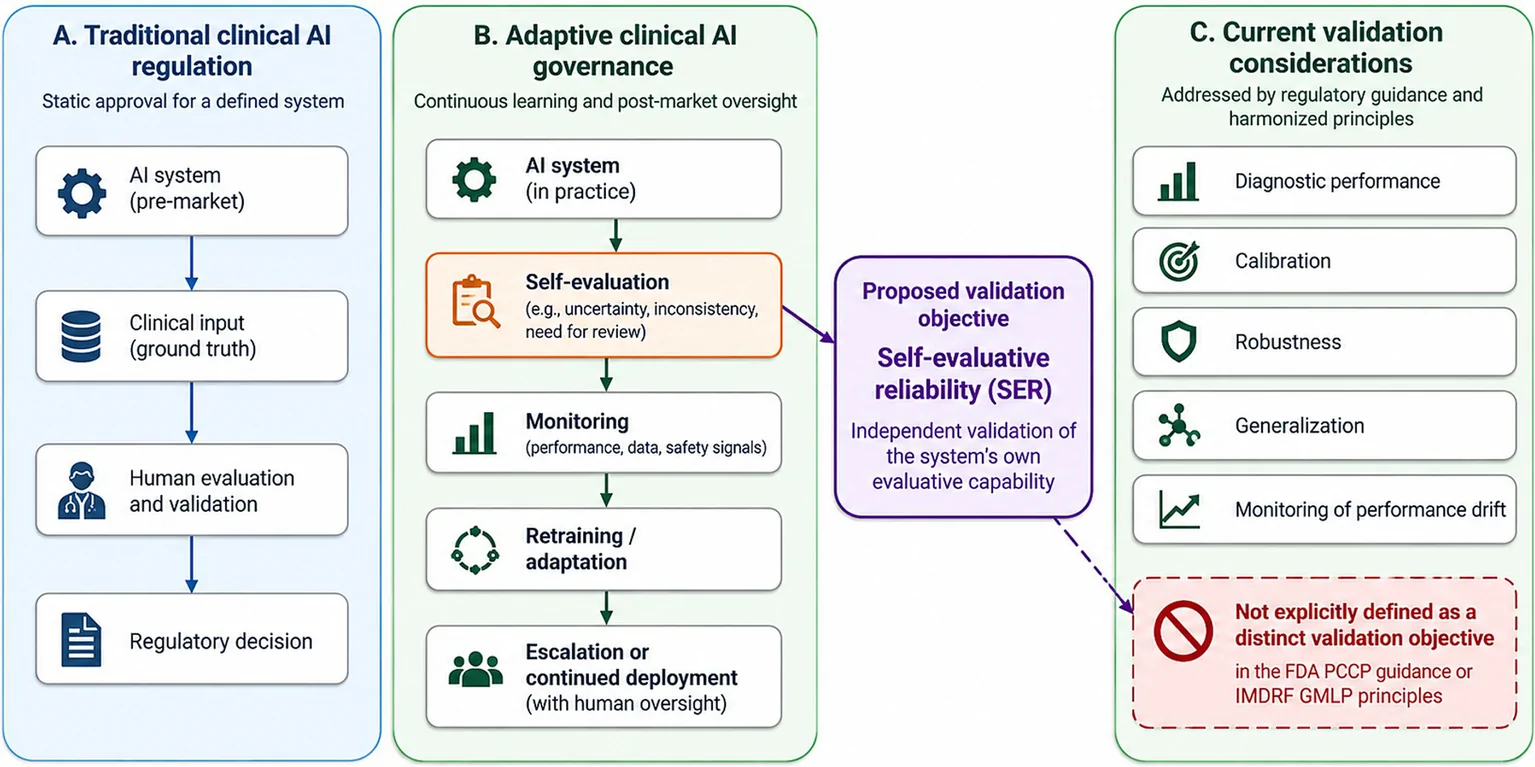
Self-evaluative reliability as a missing safety property in adaptive clinical AI governance. Traditional clinical AI regulations primarily validate model performance before regulatory approval through human evaluation. Adaptive governance extends this process by permitting AI systems to contribute to post-deployment safety assurance through self-assessment, such as identifying uncertainty, inconsistencies, or situations requiring additional review. These self-evaluative signals may subsequently inform monitoring, retraining, escalation, or continued deployment. Current regulatory guidance addresses established performance characteristics, such as diagnostic performance, calibration, robustness, and the monitoring of performance drift. However, the FDA PCCP guidance and IMDRF GMLP principles do not explicitly define validation of the AI system’s own self-evaluative capability as distinctive objective. The figure illustrates the central thesis of this Perspective: Self-evaluative reliability (SER) should be recognized as an independent safety property and explicitly validated before self-evaluation is incorporated into adaptive clinical AI governance.

BOX 1 **Definition of self-evaluative reliability (SER)**

Self-evaluative reliability (SER): the extent to which an AI system’s evaluation of its own output is both reproducible under predetermined evaluation conditions and valid against an independent reference standard. SER therefore encompasses two distinct dimensions: reliability, referring to repeatability or reproducibility of self-evaluative judgment, and validity, referring to their agreement with an appropriate external reference.

- Reliability: whether self-evaluative judgments are reproducible when the same evaluation is repeated under comparable conditions.
- Validity: whether those judgments correspond to an independent reference standard. A consistently reproduced but incorrect self-evaluation may therefore be reliable in a statistical sense but does not establish SER as a validated safety property.

Distinction from related but different concepts:

- Confidence calibration/uncertainty quantification: whether a stated probability matches with empirical accuracy, rather than whether the system accurately judges its own reasoning.
- Self-consistency: the internal coherence of outputs generated by a system across repeated queries; this coherence can be high even when the system consistently produces erroneous results.
- Inter-rater agreement: a statistical property of any set of evaluators, human or AI, whereas SER specifically concerns an AI system evaluating its own output.

In the context of this Perspective, self-evaluation refers to an evaluative process generated within the operational AI system and applied to that system’s own output. This process may encompass a second inference pass by the same model or a dedicated critic or verifier component operating within the same system architecture. A fully independent external model used solely to evaluate another system is considered AI-as-evaluator rather than self-evaluation. Such architectures may have different dependencies and failure modes, and SER should therefore be validated for the specific self-evaluation architecture in which it is used.

## 2 The missing validation objective

PCCP provides a regulatory pathway for management of anticipated modifications to AI-enabled software functions without requiring a new premarket submission for each predefined change. A PCCP specifies the planned modifications, the methods used for their development, validation, and implementation, and an assessment of their potential impacts (U.S. Food and Drug Administration (FDA), 2025). Similarly, GMLP principles emphasize monitoring the performance of deployed models and managing risks associated with retraining and modification (International Medical Device Regulators Forum (IMDRF), 2025). These principles increasingly operate within the framework of a broader lifecycle approach to clinical AI, wherein post-deployment monitoring processes can inform decisions regarding intervention, modification, retraining, or continued use (Keyes et al., 2026). These documents provide regulatory recommendations and internationally harmonized principles for monitoring performance, managing retraining risks, and evaluating predefined changes. PCCPs are becoming increasingly prominent in regulatory practice, even though public reporting and implementation approaches remain heterogeneous (Carvalho et al., 2025; Dabek et al., 2026). The unresolved issue addressed here is more narrowly focused. Neither the FDA PCCP guidance nor the IMDRF GMLP principles explicitly define the reliability of AI-generated self-assessment as a distinct validation objective in cases where such signals contribute to post-deployment surveillance. In other words, validating the clinical performance of a modified system is not equivalent to validating the reliability of a system that is itself involved in assessing the reliability of its own outputs.

This distinction separates SER from two related areas of regulatory concern. The first is whether an AI system’s clinical performance changes after deployment, including changes in discrimination, calibration, robustness, or other task-specific outcomes. The second is whether a modification falls within an authorized change protocol or requires additional regulatory review. SER addresses a prior question: if an AI-generated self-evaluative signal is used to inform either process, has the reliability of that signal itself been established? Measuring how accurately a monitoring signal detects changes in clinical performance is meaningful only after the mechanism generating that signal has been shown to be sufficiently reliable for its intended use.

## 3 Evidence that evaluative reliability deserves scrutiny

Evidence directly examining self-evaluative reliability in clinical AI remains limited. However, a growing literature on AI-based evaluation provides relevant evidence about whether evaluative judgments generated by LLMs are stable across evaluators, tasks, and evaluation settings. Although this evidence arises from AI-as-evaluator paradigms rather than direct self-evaluation, it provides an empirical basis for examining whether evaluative reliability can safely be assumed (Byun et al., 2026; Williams et al., 2026).

A smaller but more directly relevant literature has begun to examine model-generated self-assessment and self-checking in medical tasks. Boie et al. (2026) evaluated self-reported confidence across 12,000 medical question-and-answer responses, finding that confidence calibration varied substantially across models and clinical specialties, with high task accuracy not necessarily corresponding to reliable uncertainty estimation. Ryan et al. (2026) examined LLM-based fact-checking and consistency checking of healthcare responses in comparison with clinical expert assessment, demonstrating that model-generated self-checks can show substantial agreement with external references while remaining imperfect. Although these studies do not constitute a comprehensive validation of SER, they provide more direct evidence that self-generated evaluative signals can be empirically compared with independent reference standards.

Byun et al. (2026) evaluated clinical reasoning generated by three LLMs across 1,000 MIMIC-IV hospital-stay cases, with evidence–conclusion coherent independently judged by three LLM verifiers. Inter-verifier agreement on coherence was low, with Fleiss’ *κ* values ranging from 0.087 to 0.223. Similarly, Williams et al. (2026) compared LLM judges with human evaluators in global health, and they identified model-dependent differences in evaluative behavior, with agreement varying across evaluation criteria. Taken together, these findings indicate that AI-generated evaluative judgments can depend materially on the model performing the evaluation and the aspect of performance being judged.

Crucially, poor agreement is not universal. Some evaluation settings have achieved substantially higher agreement between AI-based evaluators and reference assessments, indicating that reliable automated evaluation may be achievable under appropriate task definitions, evaluator selection, and evaluation protocols (Croxford et al., 2025). This heterogeneity is itself informative. Therefore, the relevant question is not whether AI-generated evaluation is inherently reliable or unreliable, but rather under what conditions its reliability suffices for a defined governance role. More broadly, repeated-run evaluations of medical LLMs have shown that strong average performance can coexist with variability across repeated assessments, reinforcing the distinction between performance and reliability as separate properties (Zhang et al., 2026). Table 1 summarizes the principal studies informing this argument and highlights the heterogeneity of evaluative reliability across clinical AI settings.

**Table 1:** Selected empirical evidence on evaluative reliability in clinical AI and its relevance to self-evaluative reliability (SER).

Clinical evaluation setting	Evaluation paradigm	Main reliability finding	Relevance to SER	Limitation for inference to SER	Study
Clinical reasoning: Coherence	Three LLM verifiers	Low agreement; Fleiss’ *κ* 0.087–0.223	AI evaluator variability	External evaluation	(Byun et al., 2026)
Global health GenAI evaluation	LLM vs. human evaluators	Agreement varied by model and criterion	Model-dependent variability	External evaluation	(Williams et al., 2026)
Treatment-plan evaluation	AI vs. human evaluators	Systematic rater-dependent differences	Evaluator-dependent judgments	External evaluation	(Sengupta and Panda, 2026)
Clinical summarization	LLM-based evaluation	ICC up to 0.818	High reliability is achievable	External evaluation	(Croxford et al., 2025)

External evaluation indicates AI-as-evaluator or AI-human evaluator studies rather than direct evaluation of a model’s own outputs. LLM, large language model; ICC, intraclass correlation coefficient.

These findings should not be interpreted as evidence that current AI systems categorically lack the capacity for self-assessment. Disagreements may stem from differences in calibration, evaluation criteria, prompting, model architecture, or the clinical framework applied by the evaluator. Agreement does not guarantee accuracy, either. Multiple evaluators can consistently arrive at the same erroneous judgment. Therefore, the evidence supports a narrower conclusion: evaluative reliability is variable and context-dependent, and it must be measured rather than inferred from general model performance. For self-evaluation to make a meaningful contribution to post-deployment governance, both the reproducibility and the validity of the resulting judgments require independent evaluation.

Most of the current empirical bridge to SER remains inferential rather than direct. External evaluator studies demonstrate that AI-generated evaluative judgments can vary across models, criteria, and evaluation conditions. Although these studies do not prove that a model will exhibit the same behavior when evaluating its own outputs, they show why evaluation competence cannot be presumed to transfer automatically to self-evaluation. Although emerging studies on self-confidence and self-checking provide closer analogs, they assess only specific components of self-evaluation and do not yet establish SER across adaptive clinical AI systems. Direct studies in which models evaluate their own outputs against independent reference standards are therefore needed to establish SER.

## 4 Reframing self-evaluation as a safety property

Reliability and performance address different questions. A clinical AI system may achieve high average task performance while simultaneously producing unstable evaluative judgments across repeated evaluations or varying assessment conditions. Conversely, consistent self-evaluation does not establish the accuracy of those judgments. Therefore, SER should not be treated as a surrogate of clinical accuracy. It is a distinct property concerning whether an AI system’s self-evaluative judgments are sufficiently reproducible and reliable for their intended role. This distinction is particularly important when those judgments contribute to safety-critical actions such as escalation, retraining, or continued deployment (Zhang et al., 2026).

Clinical validation already acknowledges that performance alone is insufficient when a measurement cannot be reliably reproduced. Repeatability, reproducibility, and inter-rater agreement are assessed separately from diagnostic accuracy because they characterize different attributes of a measurement process. The same principle should apply when an AI system generates judgments regarding the quality or limitations of its own outputs. A system that performs well on average cannot be considered reliable in terms of self-evaluation if it yields materially different judgments when the same evaluation task is repeated under comparable conditions. Therefore, rather than presenting reliability as an entirely new regulatory concept, SER extends an established validation principle to a newly emerging form of AI-generated measurement (Tian et al., 2026).

SER must also be distinguished from self-consistency. Self-consistency methods assess whether repeated outputs from a model converge on the same answer and can be useful for estimating uncertainty or improving reliability. Recent studies have explored consistency-based approaches for certifying AI agent behavior under repeated sampling (Mouzouni, 2026). However, output consistency does not prove the validity of self-evaluation; a system can repeatedly generate the same erroneous judgment regarding its own performance. Therefore, rather than inferring validity solely from output consistency, SER requires an independent evaluation of the system’s evaluative judgments.

This distinction becomes increasingly significant as clinical AI moves toward agentic architectures. Frameworks for agentic clinical decision support distinguish planning, action, reflection, and memory as functional components rather than treating model output as a single computational event (Dinc and Ardic, 2026). In such architectures, reflective or self-assessment processes can influence subsequent actions, memory updates, or escalation decisions. SER addresses a narrower question of validation: it asks whether the evaluative judgments produced by these processes are sufficiently reliable for the role assigned to them. Therefore, the mere presence of a reflective component does not indicate a reliable self-evaluation.

The practical consequences of relying on unverified self-evaluation can be significant. An unreliable self-evaluative signal might provide a false sense of security when performance deteriorates, lead to unnecessary retraining despite the absence of meaningful degradation, or fail to identify outputs requiring human review. In adaptive or agentic systems, these errors can extend beyond a single prediction because an incorrect self-evaluative judgment may influence subsequent reasoning, tool use, memory updates, escalation, or model adaptation. This creates the potential for cascading error, in which an unreliable judgment becomes an input to later processes rather than remaining confined to the output in which it originated. Establishing SER can help define when self-evaluative signals are sufficiently dependable to inform such downstream processes and when independent evaluation or human oversight remains necessary.

## 5 Implications: validation before it becomes load-bearing

We propose that AI-generated self-evaluation become an explicit validation objective in contexts where it contributes to the management of adaptive clinical AI. This proposal does not introduce a new regulatory framework: Rather, it extends established validation principles to a capability that can increasingly contribute to post-deployment monitoring (U.S. Food and Drug Administration (FDA), 2025). Before self-evaluative judgments influence monitoring, adaptation, escalation, or continued deployment, their reliability must be demonstrated using predefined assessment protocols and appropriate reference standards.

In practice, SER validation may encompass four complementary elements. First, self-evaluative judgments should be compared against predefined reference standards across representative clinical tasks, using agreement measures appropriate to the study design. Second, acceptable reliability thresholds must be established *a priori*, based on the intended role of the self-evaluation and the clinical consequences of an unreliable decision. Third, SER should be reassessed following changes that could affect self-evaluative behavior, including relevant modifications implemented under a PCCP (U.S. Food and Drug Administration (FDA), 2025). Fourth, results should be documented as a distinct component of lifecycle validation, alongside established metrics such as clinical performance, calibration, robustness, and post-deployment drift.

It is unlikely that a single statistical metric will be suitable for all SER applications. For continuous self-evaluative scores, such as ratings of output quality or uncertainty, reliability may be assessed using intraclass correlation coefficients. Categorical judgments, such as whether an output requires escalation, may be evaluated using kappa statistics or agreement rates, whereas probabilistic estimates of correctness or uncertainty may require calibration measures. Metric selection should therefore be predetermined and justified according to the intended evaluation function, outcome type, study design, and reference standard.

As an illustrative example, consider an AI system that generates a clinical recommendation and subsequently assigns a self-evaluative judgment indicating whether human escalation is required. SER validation could repeatedly present the same set of representative outputs to the self-evaluation process and then compare the resulting escalation judgments with an independently defined clinical reference standard. Agreement and accuracy measures could be reported overall, across clinically relevant subgroups, or under distribution shift. An acceptance threshold should be specified prospectively according to the intended use and the consequences of missed or unnecessary escalation.

This perspective does not provide a comprehensive regulatory framework nor propose a universal statistical method for measuring SER. Instead, it establishes a validation objective that becomes significant in contexts where AI-generated self-evaluation contributes to post-deployment safety assurance. Defining appropriate evaluation tasks, reference standards, reliability thresholds, and reporting requirements will necessitate regulatory and clinical consensus, as well as prospective methodological studies across diverse clinical AI architectures and use cases.

While the governance of adaptive clinical AI is still evolving, there is an opportunity to establish SER as an explicit validation objective. Expecting self-evaluative behavior to be routinely incorporated into monitoring, adaptation, or deployment decisions carries the risk of an unverified capability having operational consequences before its reliability is understood. Defining SER requirements at an earlier stage will allow evidentiary standards to evolve alongside the technologies they aim to govern. Self-evaluation should become load-bearing in clinical AI governance only after its reliability has been demonstrated.

## Data Availability

The original contributions presented in the study are included in the article/supplementary material, further inquiries can be directed to the corresponding author.
